# Draft Genome Sequence of *Pseudarthrobacter phenanthrenivorans* Strain MHSD1, a Bacterial Endophyte Isolated From the Medicinal Plant *Pellaea calomelanos*

**DOI:** 10.1177/1176934320913257

**Published:** 2020-04-06

**Authors:** Khuthadzo Tshishonga, Mahloro Hope Serepa-Dlamini

**Affiliations:** Department of Biotechnology and Food Technology, Faculty of Science, University of Johannesburg, Johannesburg, South Africa

**Keywords:** *Pseudarthrobacter phenanthrenivorans*, bacterial endophyte, genome sequencing, *Pellaea calomelanos*, phylogenomic analysis

## Abstract

*Pseudarthrobacter phenanthrenivorans* strain MHSD1 is a bacterial endophyte isolated from sterilized leaves of *Pellaea calomelanos*, a medicinal plant capable of growing in arid environments. Here, we report the draft genome sequence and annotation of this bacterial endophyte. The draft genome sequence of *P. phenanthrenivorans* strain MHSD1 has 4 450 468 bp with a G + C content of 65.30%. The National Center for Biotechnology Information Prokaryotic Genome Annotation Pipeline identified a total of 4004 protein-coding genes, 56 genes coding for RNAs, and 82 pseudogenes. Biosynthesis pathways for various phytohormones such as auxin, salicylic acid, ethylene, cytokinin, jasmonic acid, abscisic acid, and gibberellins were identified. Putative genes involved in various characteristics of bacterial endophyte lifestyle such as transport, motility, adhesion, membrane proteins, secretion and delivery systems, plant cell wall modification, and detoxification were identified. Phylogenomic analysis showed *P. phenanthrenivorans* strain MHSD1 to be a subspecies of *P. phenanthrenivorans* Sphe3.

## Introduction

Endophytes are microorganisms, often bacteria or fungi, that are associated with plant tissues without causing any harm.^1^ These microorganisms can spend part or all of their life cycle within their plant hosts.^2^ In their relations with plants, they display various interactions that involve mutualism and antagonism but rarely parasitism.^3^ All plants are probably associated with endophytes.^2,4^ Endophytes promote plant growth by enhancing plant’s uptake of nutrients such as nitrogen, phosphate, and potassium; also, they biologically control plant pathogens as well as the production of secondary metabolites with pharmaceutical or biotechnological interest, and phytostimulation through the production of phytohormones^5-8^

Endophytes produce bioactive secondary metabolites; moreover, endophytes associated with medicinal plants are known to produce similar secondary metabolites as their plant host, with increased therapeutic potential.^9,10^ Thus, endophytes are alternate sources of bioactive secondary metabolites, as it is better to scale up the microbial fermentation process to increase the production of biologically active compounds, than use high amounts of plant materials, which can result in deforestation, decreased biodiversity, and conservation.^9,11^ As such, the prospects of isolating and identifying new endophyte species from plants can be beneficial. In a recent study, we isolated and identified bacterial endophytes associated with *Pellaea calomelanos*,^12^ a medicinal plant capable of growing in arid conditions.

*Pellaea calomelanos* is a fern that belongs to *Pteridaceae* family.^13^ The plant has healing properties for ailments such as asthma, head colds, coughs, and chest colds.^14,15^ One of the isolated bacterial endophytes was identified as *Arthrobacter* sp. MSHD1 using 16S ribosomal RNA (rRNA) gene sequence and biochemical characterization.^12^ The whole genome of this strain has been sequenced and the sequence data submitted to National Center for Biotechnology Information (NCBI). The draft genome sequence is described here.

## Materials and Methods

### Genomic DNA isolation, library preparation, and sequencing

Total genomic DNA was extracted from glycerol stock cultures, maintained on nutrient agar at 30°C for 48 hours using the Nucleospin Microbial DNA extraction kit as per the manufacturer’s protocol. The concentration and quality of isolated DNA were determined using the NanoDrop ND-2000 UV-Vis spectrophotometer. The DNA was sent to a commercial service provider, Agricultural Research Council, Onderstepoort, South Africa, for sequencing with Illumina MiSeq platform. Briefly, the library was prepared using NEBNextUltra II DNA kit following the manufacturer’s protocol with a paired-end sequencing strategy (300 bp insert size) using Illumina MiSeq instrument v3.

### Pre-processing, genome assembly, and annotation

All pre-annotation analyses were performed on Galaxy web platform available at https://usegalaxy.org.^16^ FastQC v 0.69 was used to assess the quality of the raw reads.^17^ Using default parameters, the sequence reads were *de novo* assembled using Unicycler v 0.4.1.1^18^ and assessed with Quast v 4.6.3.^19^ The draft genome sequence was submitted to NCBI and annotated using Prokaryotic Genome Annotation Pipeline (PGAP)^20^ and Rapid Annotations using Subsystems Technology (RAST) server.^21-23^

### Bioinformatics

The phylogenomic analysis was undertaken with the Type Strain Genome Server (TYGS) available at https://tygs.dsmz.de/^24^ and OrthoANI (Orthologous Average Nucleotide Identity) with the Orthologous Average Nucleotide Identity Tool (OAT) software.^25^ The genomic islands (GI) were identified by screening the PGAP annotation file generated from NCBI on the IslandViewer 4 Web site (http://www.pathogenomics.sfu.ca/islandviewer/).^26^ The Clustered Regularly Interspaced Short Palindromic Repeats (CRISPR) were predicted by CRISPRCas finder software.^27-29^ The RAST server was used to annotate and classify predicted genes according to function.^21-23^ The shared and unique genes of MHSD1 were analyzed by comparing it with 1 bacterial endophyte genome (*Arthrobacter* sp. PAMC 25486) as well as 3 closely related genomes (*Pseudarthrobacter chlorophenolicus* A6, *P. phenanthrenivorans* Sphe3, *P. sulfonivorans* Ar51) using EDGAR 2.0.^30^ The genome was masked for repeats using RepeatMasker.^31^

### Accession of the genome sequence

The data from this Whole Genome Shotgun project have been deposited at DDBJ/ENA/GenBank with BioProject number PRJNA549841 and BioSample number SAMN12098155 under the accession VHJD00000000. The version described here is VHJD01000000.

## Interpretation of Data Set

**Figure 1.**
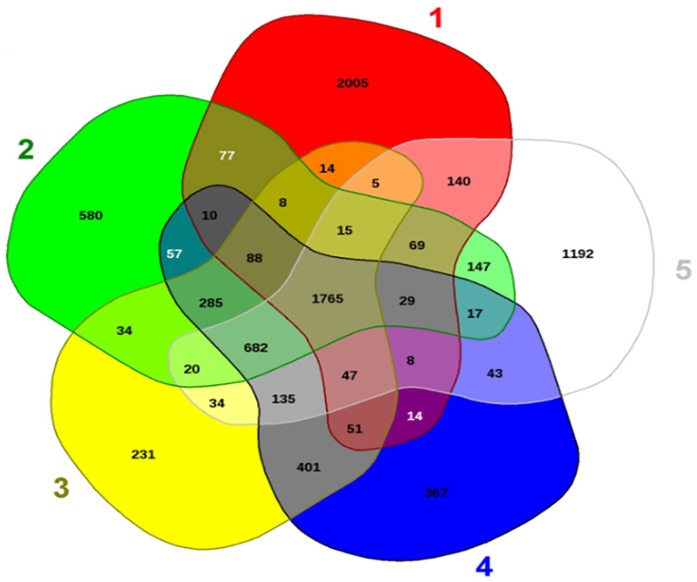
Venn diagram of shared and unique genes of *Pseudarthrobacter phenanthrenivorans* MHSD1 and selected comparison species. 1: *Arthrobacter* sp. PAMC 25486; 2: *Pseudarthrobacter chlorophenolicus* A6; 3: *P phenanthrenivorans* Sphe3; 4: *P. phenanthrenivorans* MHSD1; 5: *Pseudarthrobacter sulfonivorans* Ar51.

**Figure 2.**
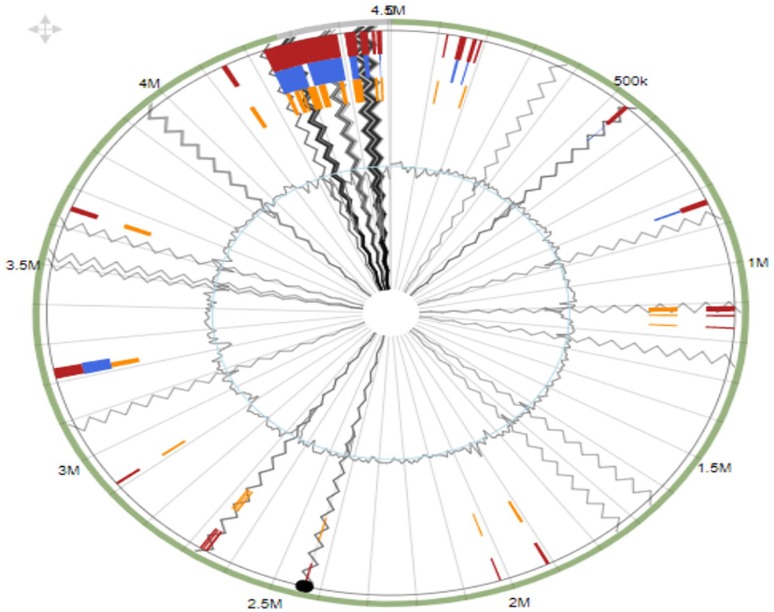
Genetic islands of *Pseudarthrobacter phenanthrenivorans* MHSD1 aligned with reference genome of *P phenanthrenivorans* Sphe3. A total of 24 genetic islands were predicted using IslandViewer 4. The green outer circle represents the scale line of the genome in Mbps, and the obtained genomic islands are represented by the following colors: IslandPath-DIMOB (blue), SIGI-HMM (orange), and integrated detection (red). Gray lines indicate contig boundaries.

The draft genome of strain MHSD1 had 56 contigs with a total length of 4 450 468 bp, G + C content of 65.30%, and an *N50* value of 363 437 bp. Using PGAP, the predicted number of genes was 4142, of which 4004 of them were protein-coding genes (CDSs), 56 were RNAs, 82 were pseudogenes, and 3 were non-coding RNAs (ncRNAs). The predicted RNA coding genes include 50 transfer RNAs (tRNAs) and 3 rRNAs (5S, 16S, and 23S). A total of 8566 bp comprising 0.19% were masked. The genome features are in Table 1. The TYGS whole genome–based taxonomic analysis showed that MHSD1 was closely related to *Pseudarthrobacter phenanthrenivorans* strain Sphe3 as shown in Figures 1 and 2 (Supplementary Data). MHSD1 formed a sister clade with Sphe3 in both phylogenetic trees.

**Table 1.** Genome features of *Pseudarthrobacter phenanthrenivorans* strain MHSD1.

Attribute	Value
Genomic size (bp)	4,450,468
GC content	65.30%
Total number of genes	4142
Protein coding genes	4004
Number of RNAs	56
rRNA genes	3
tRNA gene	50
Protein coding genes with function prediction	3553
CRISPR repeats	1

Abbreviations: CRISPR, Clustered Regularly Interspaced Short Palindromic Repeats; rRNA, ribosomal RNA; tRNA, transfer RNA.

**Figure 3.**
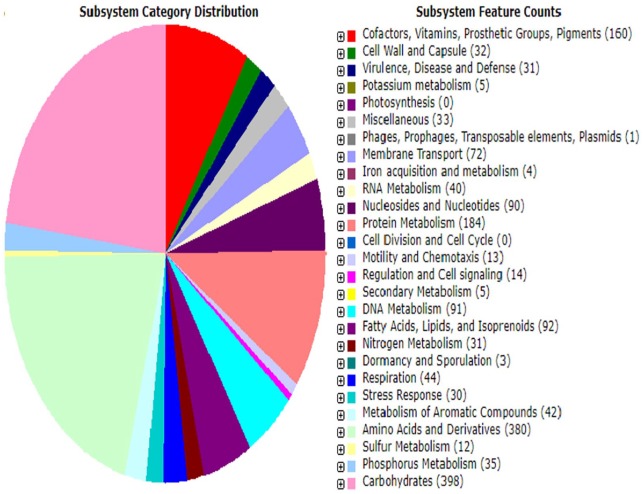
Functional classification of predicted genes of *Pseudarthrobacter phenanthrenivorans* MHSD1 genome based on RAST annotation server. RAST indicates Rapid Annotations using Subsystems Technology.

MHSD1 had a digital DNA-DNA hybridization (dDDH) of 85.3% and G + C% content difference of 0.03 with *P. phenanthrenivorans* Sphe3 (Table 1, Supplementary Data). The observed dDDH of 85.3% was greater than the species boundary value of dDDH >70% for delineating bacterial species as closely related species^32^; in addition, this value exceeded the dDDH >79%-80% for delineating subspecies.^24^ Based on the TYGS phylogenomic classification, MHSD1 is delineated as a subspecies of *P. phenanthrenivorans* Sphe3; initial identification of MSHD1 using the 16S rRNA gene was *Arthrobacter* sp. strain MSHD1. MHSD1 showed lower OrthoANI values (<70%) (Figure 3, Supplementary Data) than the species boundary of >95%-96%.^25^ Although the OrthoANI values were lower, the delineation of MHSD1 as a subspecies of *P. phenanthrenivorans* Sphe3 was based on the dDDH value in the TYGS because it was an enhanced method for species delineation and facilitates classification and identification of species as well as subspecies by comparison with published and described type strains.^24^

*Pseudarthrobacter phenanthrenivorans* strain MHSD1 shared 682 common genes with other closely related *Pseudarthrobacter* species, and only 14 genes were shared with bacterial endophyte *Arthrobacter* sp. PAMC 25486 (Figure 1). A total of 1765 genes were common among MHSD1 and all the selected comparison species (Figure 1). The 14 exclusive common genes between MHSD1 and PAMC 25486 (results not shown) encode transport proteins and transcriptional regulators, which are essential bacterial endophyte genes that have been previously identified in other bacterial endophyte species.^33,34^ *Pseudarthrobacter phenanthrenivorans* strain MHSD1 had 367 unique genes, whereas *P. phenanthrenivorans* strain Sphe3 had 231 unique genes; this can be attributed to MHSD1 having a larger genome length than Sphe3.

*Pseudarthrobacter phenanthrenivorans* strain MHSD1 was found to consist of several sets of genes acquired through horizontal gene transfer. As such, 24 GI (Figure 2) were identified in *P. phenanthrenivorans* strain MHSD1 genome when aligned to reference genome *P. phenanthrenivorans* Sphe3.^35^ The details of the genes clustered on the genomic islands are shown in Table 2 (Supplementary Data). We identified only one CRISPR system with 1 spacer and 11 repeats (Table 2). Functional classification of the genes in *P. phenanthrenivorans* strain MHSD1 based on RAST annotation (Figure 3) showed that most of the predicted genes are involved in carbohydrate metabolism, which is consistent with the bacterial endophyte lifestyle within which acquisition and mobilization of nutrients such as phosphate, nitrogen, and iron are important for symbiotic plant-bacteria interaction.^36^

**Table 2.** Clustered Regularly Interspaced Short Palindromic Repeats (CRISPR) sequences present within *Pseudarthrobacter phenanthrenivorans* strain MHSD1 identified using CRISPRCasFinder.

Element	CRISPR ID/cas type	Start	End	Gene spacer	Repeat consensus/cas gene	Direction	Evidence level
CRISPR	Contig_4_1	84903	85015	1	ACCGGCTGCGGGACGCGG ACCACCGGGACGGGGACC ACCGGCACCGGCTGCGGG ACGCGGACCACCGGGACG GTCGCCGTCGGTGCGGCTT CCACCCGGACGGGGCATG CCCTGGGACGTGGCGAAC GGGTTGTTGCCGGGACGG GGAGCACGCTCGCCGTCC CCGCCGCGGCCGCGGGGC ATGCCCTGGGACGTGGCG AAGGGGTTGTTGCCCGGG CGGGGACCGCCGGGGCGG GGAGCTGAGCCACCCTGT GATCCGCCGGAGCGGGTG GGTGCTGCAGGGGTTTCA GCTTTGGGGGCCGGCCGT GCGCCG	ND	1

Abbreviation: ND, not determined.

In this study, several putative genes involved in bacterial endophyte behavior or lifestyle were predicted and compared with bacterial endophyte *Enterobacter* sp. 638 as well as nonendophyte *P. phenanthrenivorans* Sphe3 (Table 3, Supplementary Data). Genes putatively involved in transport, motility, adhesion, membrane proteins, secretion and delivery systems, plant cell wall modification, detoxification, substrate utilization, stress protection, and transcriptional regulators were identified. In addition, genes important in bacterial endophyte life style, such as those involved in nitrogen fixation and siderophore production, were identified in MHSD1. Although most of the genes present in bacterial endophytes, *Enterobacter* sp. 638 and *P. phenanthrenivorans* MHSD1, were also present in *P. phenanthrenivorans* Sphe3, distinctness of the bacterial endophytes was due to the presence of genes encoding transport proteins and transcriptional regulators important in endophytic behavior or lifestyle, which were not present in the latter. More work is currently underway to describe MHSD1 as a subspecies of *P. phenanthrenivorans* Sphe3.

Biosynthesis pathways of various phytohormones of *P. phenanthrenivorans* MHSD1 consist of various important plant hormones such as auxin, salicylic acid, ethylene, cytokinin, jasmonic acid, abscisic acid, and gibberellins (Figure 4, Supplementary Data). The phytohormones are essential for the development and growth of plants through various mechanisms such as cell elongation, division and differentiation, access to nutrients, stress tolerance, and defense against phytopathogens.^37-39^

## Supplemental Material

supplementary_data_final_EB_xyz30819af645758_(1) – Supplemental material for Draft Genome Sequence of Pseudarthrobacter phenanthrenivorans Strain MHSD1, a Bacterial Endophyte Isolated From the Medicinal Plant Pellaea calomelanos Click here for additional data file. Supplemental material, supplementary_data_final_EB_xyz30819af645758_(1) for Draft Genome Sequence of Pseudarthrobacter phenanthrenivorans Strain MHSD1, a Bacterial Endophyte Isolated From the Medicinal Plant Pellaea calomelanos by Khuthadzo Tshishonga and Mahloro Hope Serepa-Dlamini in Evolutionary Bioinformatics
