# Supplementary material for: Draft Genome Sequence of Pseudarthrobacter phenanthrenivorans Strain MHSD1, a Bacterial Endophyte Isolated From the Medicinal Plant Pellaea calomelanos
Source: Evol Bioinform Online. 2020 Apr 6;16:1176934320913257. doi: 10.1177/1176934320913257 (PMC7136937; doi:10.1177/1176934320913257)
Supplement: supplementary_data_final_EB_xyz30819af645758_(1) – Supplemental material for Draft Genome Sequence of Pseudarthrobacter phenanthrenivorans Strain MHSD1, a Bacterial Endophyte Isolated From the Medicinal Plant Pellaea calomelanos [file supplementary_data_final_EB_xyz30819af645758_(1).pdf]

Draft Genome sequence of *Pseudarthrobacter phenanthrenivorans* strain MHSD1 a bacterial endophyte isolated from medicinal plant leaves *Pellaea calomelanos*

Supplementary Data

Khuthadzo Tshishonga and Mahloro Hope Serepa-Dlamini

Department of Biotechnology and Food Technology, Faculty of Science, University of Johannesburg, Doornfontein Campus, PO Box 17011, Doornfontein 2028, Johannesburg, South Africa

* Correspondence:

Corresponding Author

hopes@uj.ac.za

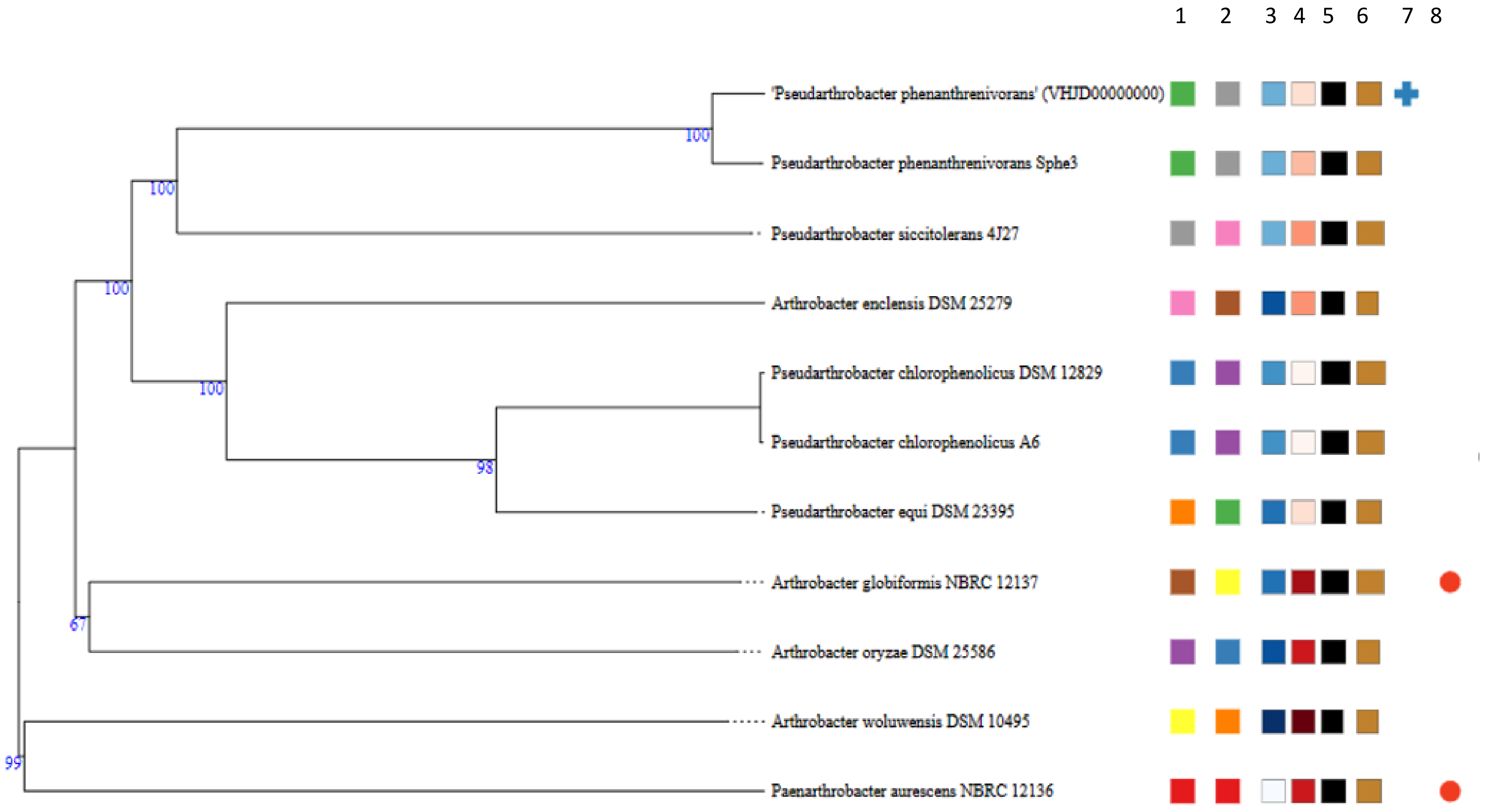

Figure 1. TYGS genome BLAST Distance Phylogeny (GBDP) tree (whole-genome sequence-based) of *Pseudarthrobacter phenanthrenivorans* strain MHSD1 and closely related species. Tree inferred with FastME 2.1.6.1 from GBDP distances calculated from genome sequences. The branch lengths are scaled in terms of GBDP distance formula d5. The numbers above branches are GBDP pseudo-bootstrap support values from 100 replications, with an average branch support of 90.6 %.The tree was rooted at the midpoint. Leaf labels are annotated by affiliation to (1) species cluster, (2) Subspecies cluster (3) percentage G+C, (4) Delta statistics, (5) Genome size, (6) Protein count , (7) User stain, (8) type species

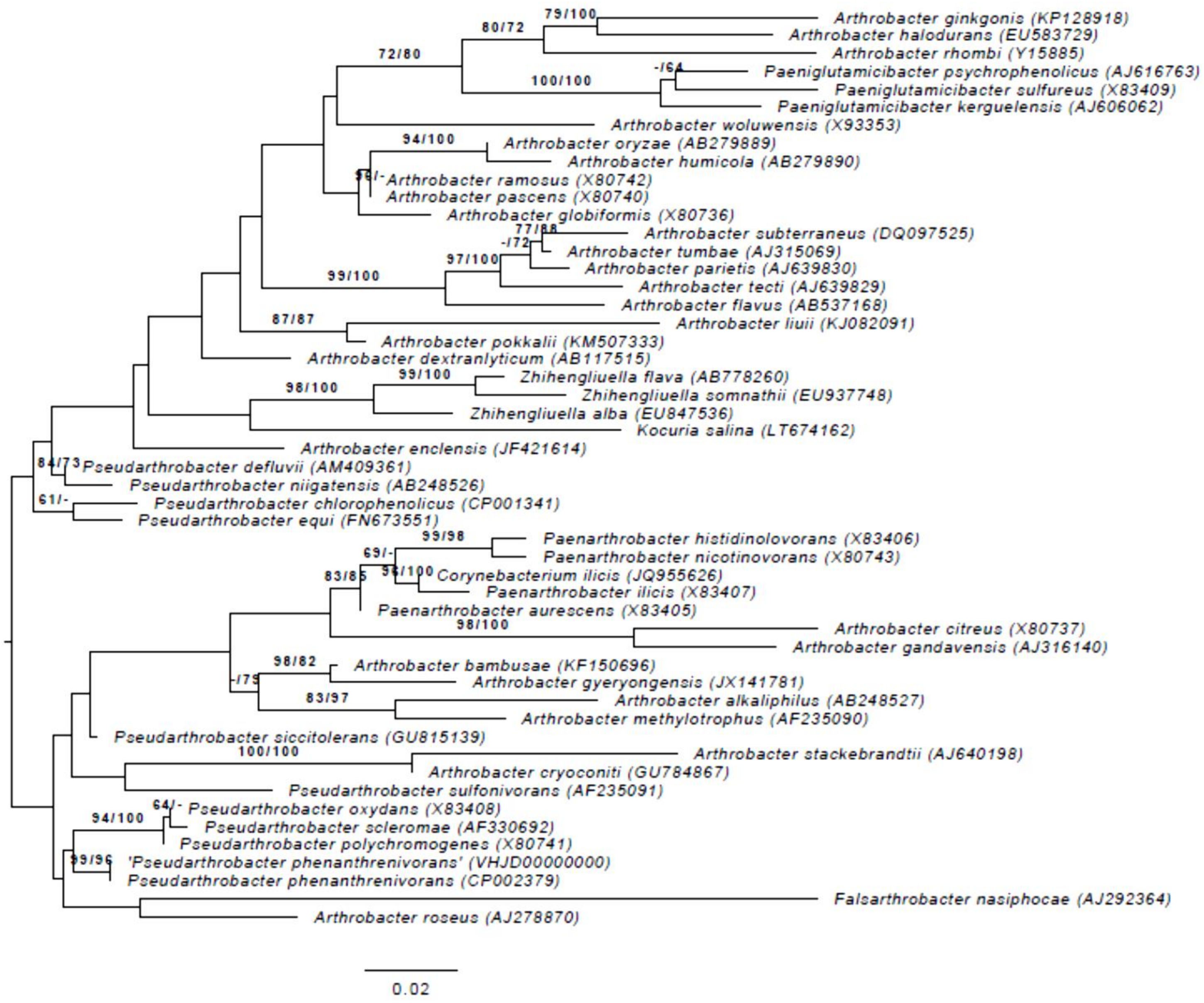
Arthrobacter ginkgonis (KP128918)
Arthrobacter halodurans (EU583729)
Arthrobacter rhombi (Y15885)
Paeniglutamicibacter psychrophenolicus (AJ616763)
Paeniglutamicibacter sulfureus (X83409)
Paeniglutamicibacter kerguelensis (AJ606062)
Arthrobacter woluwensis (X93353)
Arthrobacter oryzae (AB279889)
Arthrobacter humicola (AB279890)
Arthrobacter ramosus (X80742)
Arthrobacter pascens (X80740)
Arthrobacter globiformis (X80736)
Arthrobacter subterraneus (DQ097525)
Arthrobacter tumbae (AJ315069)
Arthrobacter parietis (AJ639830)
Arthrobacter tecti (AJ639829)
Arthrobacter flavus (AB537168)
Arthrobacter liuii (KJ082091)
Arthrobacter pokkalii (KM507333)
Arthrobacter dextranlyticum (AB117515)
Zhihengliuella flava (AB778260)
Zhihengliuella somnathii (EU937748)
Zhihengliuella alba (EU847536)
Kocuria salina (LT674162)
Arthrobacter enclensis (JF421614)
Pseudarthrobacter defluvii (AM409361)
Pseudarthrobacter niigatensis (AB248526)
Pseudarthrobacter chlorophenolicus (CP001341)
Pseudarthrobacter equi (FN673551)
Paenarthrobacter histidinolovorans (X83406)
Paenarthrobacter nicotinovorans (X80743)
Corynebacterium ilicis (JQ955626)
Paenarthrobacter ilicis (X83407)
Paenarthrobacter aurescens (X83405)
Arthrobacter citreus (X80737)
Arthrobacter gandavensis (AJ316140)
Arthrobacter bambusae (KF150696)
Arthrobacter gyeryongensis (JX141781)
Arthrobacter alkaliphilus (AB248527)
Arthrobacter methylotrophus (AF235090)
Pseudarthrobacter siccitolerans (GU815139)
Arthrobacter stackebrandtii (AJ640198)
Arthrobacter cryoconiti (GU784867)
Pseudarthrobacter sulfonivorans (AF235091)
Pseudarthrobacter oxydans (X83408)
Pseudarthrobacter scleromae (AF330692)
Pseudarthrobacter polychromogenes (X80741)
'Pseudarthrobacter phenanthrenivorans' (VHJD00000000)
Pseudarthrobacter phenanthrenivorans (CP002379)
Falsarthrobacter nasiphocae (AJ292364)
Arthrobacter roseus (AJ278870)
79/100
80/72
72/80
100/100
-/64
94/100
77/88
-/72
97/100
99/100
87/87
99/100
98/100
84/73
61/-
99/98
69/-
83/85
98/100
98/82
-/79
83/97
100/100
64/-
94/100
0.02

Figure 2. Maximum likelihood genome BLAST Distance Phylogeny (GBDP) tree (16S rDNA gene sequence-based) of *Pseudarthrobacter phenanthrenivorans* strain MHSD1 and closely related species, tree inferred with FastME 2.1.6.1, from GBDP distances calculated from 16S rDNA gene sequences. The branch lengths are scaled in terms of GBDP distance formula d5. The numbers above branches are GBDP pseudo-bootstrap support values from 100 replications, with an average branch support of 83.3 %. The tree was rooted at the midpoint.

Table 1. Pairwise comparisons of *Pseudarthrobacter phenanthrenivorans* strain MHSD1 vs. type strain genomes.

**Query strain**	**Subject strain**	**dDDH (d0, in %)**	**C.I. (d0, in %)**	**dDDH (d4, in %)**	**C.I. (d4, in %)**	**dDDH (d6, in %)**	**C.I. (d6, in %)**	**G+C content difference (in %)**
'Pseudarthrobacter phenanthrenivorans' (VHJD00000000)	*Pseudarthrobacter phenanthrenivorans* Sphe3	81.6	[77.7 - 84.9]	86.2	[83.5 - 88.4]	85.3	[82.1 - 88.0]	0.03
'Pseudarthrobacter phenanthrenivorans' (VHJD00000000)	*Pseudarthrobacter siccitolerans* 4J27	41.7	[38.3 - 45.1]	25.7	[23.4 - 28.2]	36.5	[33.5 - 39.6]	0.21
'Pseudarthrobacter phenanthrenivorans' (VHJD00000000)	*Pseudarthrobacter equi* DSM 23395	37	[33.6 - 40.5]	24.1	[21.7 - 26.5]	32.6	[29.6 - 35.7]	0.79
'Pseudarthrobacter phenanthrenivorans' (VHJD00000000)	*Arthrobacter enclensis* DSM 25279	39.1	[35.7 - 42.5]	23.9	[21.6 - 26.3]	34	[31.0 - 37.0]	1.76
'Pseudarthrobacter phenanthrenivorans' (VHJD00000000)	*Pseudarthrobacter chlorophenolicus* DSM 12829	34.3	[31.0 - 37.9]	23.8	[21.5 - 26.3]	30.7	[27.7 - 33.8]	0.6
'Pseudarthrobacter phenanthrenivorans' (VHJD00000000)	*Pseudarthrobacter chlorophenolicus* A6	34.4	[31.0 - 37.9]	23.8	[21.5 - 26.3]	30.7	[27.8 - 33.8]	0.62
'Pseudarthrobacter phenanthrenivorans' (VHJD00000000)	*Arthrobacter globiformis* NBRC 12137	23.6	[20.3 - 27.3]	22.6	[20.3 - 25.1]	22.4	[19.6 - 25.5]	0.87

'Pseudarthrobacter phenanthrenivorans' (VHJD00000000)	Arthrobacter oryzae DSM 25586	20.2	[17.0 - 23.8]	22.5	[20.2 - 25.0]	19.6	[16.9 - 22.7]	1.76
'Pseudarthrobacter phenanthrenivorans' (VHJD00000000)	Arthrobacter woluwensis DSM 10495	15	[12.1 - 18.4]	21.7	[19.4 - 24.1]	15.2	[12.7 - 18.0]	2.77
'Pseudarthrobacter phenanthrenivorans' (VHJD00000000)	Paenarthrobacter aurescens NBRC 12136	18	[14.9 - 21.5]	20.6	[18.4 - 23.0]	17.6	[15.0 - 20.6]	3.31

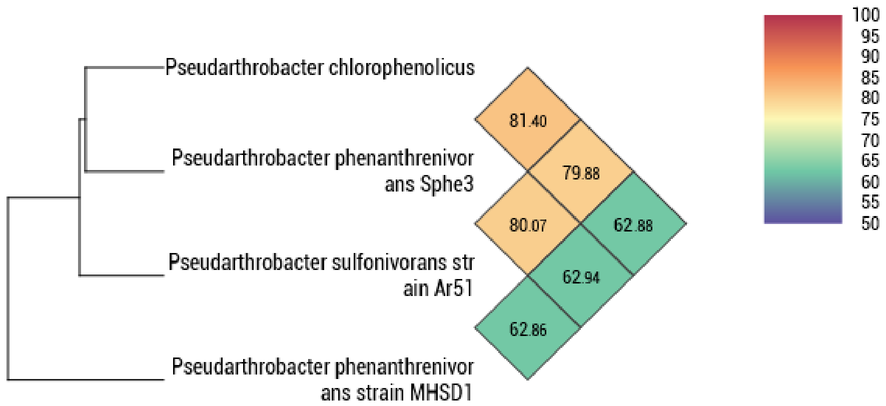

Figure 3. Heatmap generated with OAT software indicating OrthoANI values for *Pseudarthrobacter phenanthrenivorans* MHSD1 and closely related *Pseudarthrobacter* species.

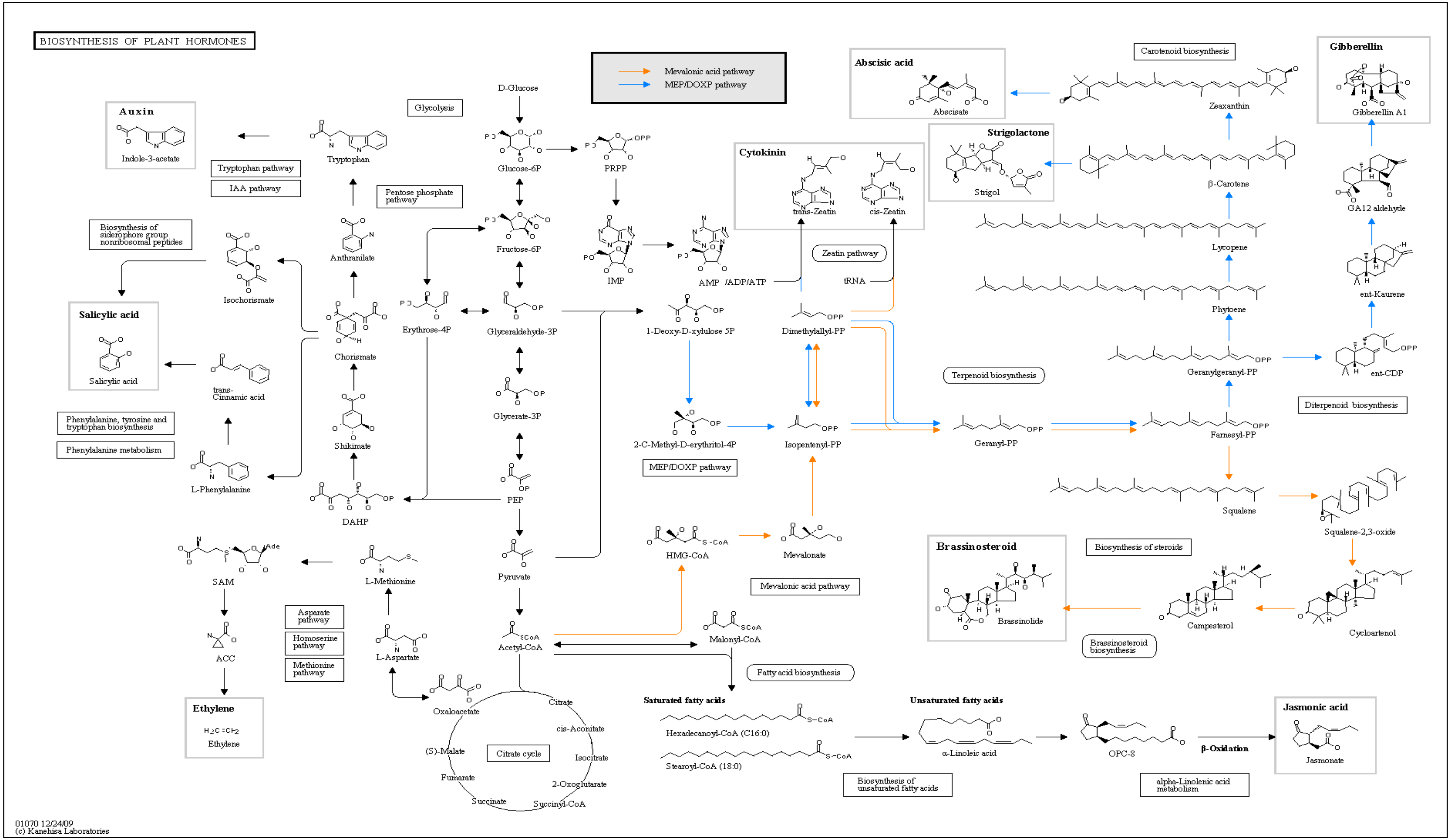

Figure 4. Biosynthesis pathways of plant hormones in *Pseudarthrobacter phenanthrenivorans* MHSD1 aligned against *Enterobacter* sp. 638. Orange arrows represent Mevalonic acid pathway and blue arrows represent MEP/DOXP pathway.

Table 2. Genomic islands genes within *Pseudarthrobacter phenanthrenivorans* strain MHSD1 aligned against reference genome within *Pseudarthrobacter phenanthrenivorans* strain Sphe3 genome. The genomic islands genes prediction methods are represented by the following colours: IslandPath-DIMOB (blue), SIGI-HMM (orange), and integrated detection (empty blocks).

Prediction Method	Gene Name	Product
	FJ661_10095	hydantoinase B/oxoprolinase family protein
	FJ661_10090	hydantoinase/oxoprolinase family protein
	solA	N-methyl-L-tryptophan oxidase
	FJ661_10080	MurR/RpiR family transcriptional regulator
	FJ661_10075	hypothetical protein
	FJ661_10070	LacI family transcriptional regulator
	FJ661_10065	ABC transporter ATP-binding protein
	FJ661_10060	sugar phosphate isomerase/epimerase
	FJ661_10000	N-acetylglucosamine-6-phosphate deacetylase
	FJ661_09995	N-acetylmannosamine-6-phosphate 2-epimerase
	FJ661_09990	ROK family protein
	FJ661_09985	dihydrodipicolinate synthase family protein
	FJ661_09980	ABC transporter ATP-binding protein
	FJ661_09975	dipeptide/oligopeptide/nickel ABC transporter permease/ATP-binding protein
	FJ661_09970	ABC transporter permease
	FJ661_09965	ABC transporter substrate-binding protein
	FJ661_09960	DUF4862 family protein
	FJ661_09955	exo-alpha-sialidase
	FJ661_09950	sialidase
	FJ661_09945	plasmid pRiA4b ORF-3 family protein
	FJ661_09940	recombinase family protein
	FJ661_09935	ROK family protein
	FJ661_09930	hypothetical protein
	FJ661_09925	dextranase
	FJ661_09920	alpha-glucosidase
	FJ661_09915	carbohydrate ABC transporter permease
	FJ661_09910	sugar ABC transporter permease
	FJ661_09905	extracellular solute-binding protein
	FJ661_09900	ROK family transcriptional regulator
	FJ661_09895	dextranase
	FJ661_09890	glucan 1,4-alpha-glucosidase
	FJ661_09890	glucan 1,4-alpha-glucosidase
	FJ661_09885	ROK family protein
	FJ661_09880	hypothetical protein
	FJ661_09875	Fis family transcriptional regulator
	FJ661_09870	helix-turn-helix transcriptional regulator

	FJ661_09865	site-specific integrase
	FJ661_09860	DUF4193 domain-containing protein
	FJ661_09855	hypothetical protein
	FJ661_09850	hypothetical protein
	FJ661_09845	hypothetical protein
	FJ661_09840	GPP34 family phosphoprotein
	ppsA	phosphoenolpyruvate synthase
	FJ661_09830	kinase/pyrophosphorylase
	FJ661_09825	hypothetical protein
	FJ661_09820	lactate utilization protein C
	FJ661_09815	iron-sulfur cluster-binding protein
	FJ661_09810	(Fe-S)-binding protein
	FJ661_09805	L-lactate permease
	FJ661_09805	L-lactate permease
	FJ661_09800	FadR family transcriptional regulator
	FJ661_09795	cytochrome d ubiquinol oxidase subunit II
	FJ661_09790	pentapeptide repeat-containing protein
	FJ661_09785	hypothetical protein
	FJ661_09780	hypothetical protein
	FJ661_09775	hypothetical protein
	FJ661_09770	recombinase family protein
	FJ661_09765	DUF4193 domain-containing protein
	FJ661_09760	Ldh family oxidoreductase
	FJ661_09755	DctP family TRAP transporter solute-binding subunit
	FJ661_17905	MFS transporter
	FJ661_17910	hypothetical protein
	FJ661_17915	LamB/YcsF family protein
	FJ661_17920	DUF445 domain-containing protein
	FJ661_17925	glycerophosphodiester phosphodiesterase
	FJ661_17930	hypothetical protein
	FJ661_17935	AzlC family ABC transporter permease
	FJ661_17940	AzlD domain-containing protein
	FJ661_17945	hypothetical protein
	FJ661_17950	hypothetical protein
	FJ661_17955	hypothetical protein
	FJ661_17960	TetR/AcrR family transcriptional regulator
	FJ661_17965	thiamine-binding protein
	FJ661_17970	O-methyltransferase
	FJ661_17975	DNA starvation/stationary phase protection protein
	FJ661_17980	CsbD family protein
	FJ661_17985	hypothetical protein

	FJ661_17990	YihY/virulence factor BrkB family protein
	FJ661_20625	phage holin family protein
	FJ661_20630	hypothetical protein
	FJ661_13650	hypothetical protein
	FJ661_13645	hypothetical protein
	FJ661_13640	NAD-dependent epimerase/dehydratase family protein
	FJ661_13635	sigma-70 family RNA polymerase sigma factor
	FJ661_13630	HAD-IIIA family hydrolase
	FJ661_13625	glycosyltransferase family 9 protein
	FJ661_13620	glycosyltransferase family 4 protein
	FJ661_13615	glycosyltransferase family 1 protein
	FJ661_12340	ExeM/NucH family extracellular endonuclease
	FJ661_12330	NAD(P)H-quinone oxidoreductase
	FJ661_12325	PadR family transcriptional regulator
	FJ661_12320	DUF1540 domain-containing protein
	FJ661_12315	carbon starvation protein A
	FJ661_12310	YbdD/YjiX family protein
	FJ661_12305	bacterial proteasome activator family protein
	FJ661_12300	hypothetical protein
	FJ661_12295	aldo/keto reductase
	FJ661_12290	hypothetical protein
	FJ661_12285	hypothetical protein
	FJ661_12280	hypothetical protein
	FJ661_12275	hypothetical protein
	FJ661_12270	DNA-binding protein
	FJ661_12265	ABC transporter permease
	FJ661_12260	hypothetical protein
	FJ661_12255	helix-turn-helix domain-containing protein
	FJ661_12250	hypothetical protein
	FJ661_12245	hypothetical protein
	FJ661_12240	hypothetical protein
	FJ661_12235	hypothetical protein
	FJ661_12230	hypothetical protein
	FJ661_12225	recombinase family protein
	FJ661_12215	alpha/beta hydrolase
	FJ661_12210	DNA polymerase III subunit delta'
	FJ661_12205	dTMP kinase
	FJ661_12200	aminotransferase class I/II-fold pyridoxal phosphate-dependent enzyme
	FJ661_12195	DUF2516 family protein
	FJ661_12190	class I SAM-dependent methyltransferase
	FJ661_12185	phosphoglyceromutase

	phoU	phosphate signaling complex protein PhoU
	FJ661_12175	two-component sensor histidine kinase
	FJ661_16000	amidase
	FJ661_16005	acetamidase
	FJ661_16010	FadR family transcriptional regulator
	FJ661_16015	MFS transporter
	FJ661_16020	Lrp/AsnC family transcriptional regulator
	hisC	histidinol-phosphate transaminase
	FJ661_16030	amino acid permease
	FJ661_16035	hypothetical protein
	FJ661_16040	hypothetical protein
	FJ661_16045	hypothetical protein
	FJ661_16050	trypsin-like peptidase domain-containing protein
	FJ661_17995	radical SAM protein
	FJ661_18000	helix-hairpin-helix domain-containing protein
	FJ661_18005	hypothetical protein
	FJ661_18010	AAA family ATPase
	FJ661_18015	DUF4234 domain-containing protein
	FJ661_18020	hypothetical protein
	FJ661_18025	hypothetical protein
	FJ661_18030	FadR family transcriptional regulator
	FJ661_18035	carbon-nitrogen hydrolase family protein
	FJ661_18040	APC family permease
	FJ661_18045	carbon-nitrogen hydrolase family protein
	FJ661_18050	SHOCT domain-containing protein
	FJ661_18055	DUF262 domain-containing protein
	FJ661_18060	aminotransferase class I/II-fold pyridoxal phosphate-dependent enzyme
	FJ661_18065	amino acid permease
	FJ661_18070	hypothetical protein
	FJ661_18075	hypothetical protein
	FJ661_18075	hypothetical protein
	FJ661_18080	hypothetical protein
	FJ661_18085	APC family permease
	FJ661_18090	carbon-nitrogen hydrolase family protein
	FJ661_18095	restriction endonuclease
	FJ661_18100	amino acid transporter
	FJ661_18105	lamin tail domain-containing protein
	FJ661_18110	hypothetical protein
	FJ661_18115	DUF3427 domain-containing protein
	FJ661_18165	MFS transporter
	FJ661_18170	nucleoside hydrolase

	FJ661_18175	NAD(P)/FAD-dependent oxidoreductase
	FJ661_18180	hypothetical protein
	FJ661_18185	hypothetical protein
	FJ661_18190	DUF4230 domain-containing protein
	FJ661_18195	HNH endonuclease
	FJ661_18200	hypothetical protein
	FJ661_18205	SRPBCC family protein
	FJ661_18210	antibiotic biosynthesis monooxygenase
	FJ661_18215	NAD(P)-dependent alcohol dehydrogenase
	FJ661_18220	iron-sulfur cluster assembly protein
	FJ661_02485	hypothetical protein
	FJ661_02480	hypothetical protein
	FJ661_02475	peroxidase-related enzyme
	FJ661_02470	serine hydrolase family protein
	FJ661_02465	hypothetical protein
	FJ661_02460	hypothetical protein
	minC	septum site-determining protein MinC
	FJ661_02450	nuclear transport factor 2 family protein
	FJ661_02445	TetR/AcrR family transcriptional regulator
	FJ661_02440	GNAT family N-acetyltransferase
	FJ661_02015	energy-coupling factor transporter transmembrane protein EcfT
	FJ661_02010	ABC transporter ATP-binding protein
	FJ661_02005	biotin transporter BioY
	FJ661_02000	methyltransferase domain-containing protein
	FJ661_01995	VOC family protein
	FJ661_01990	hypothetical protein
	FJ661_01985	hypothetical protein
	FJ661_01980	hypothetical protein
	FJ661_01975	transcription initiation protein
	FJ661_01970	LD-carboxypeptidase
	FJ661_01965	uridine kinase
	FJ661_00025	hypothetical protein
	FJ661_00015	NAD-dependent protein deacetylase
	FJ661_00010	peroxiredoxin
	FJ661_00005	DUF3052 domain-containing protein
	FJ661_20640	pyruvate dehydrogenase (acetyl-transferring), homodimeric type
	FJ661_16055	pyruvate dehydrogenase (acetyl-transferring), homodimeric type
	FJ661_16060	PucR family transcriptional regulator
	FJ661_16065	ACP S-malonyltransferase
	FJ661_17085	type II secretion system F family protein
	FJ661_17090	type II secretion system protein F

	FJ661_17095	CpaF family protein
	FJ661_17100	hypothetical protein
	FJ661_17105	glycoside hydrolase
	FJ661_17110	hypothetical protein
	FJ661_17115	DUF2142 domain-containing protein
	FJ661_17120	transposase
	FJ661_20585	IS481 family transposase
	FJ661_07225	hypothetical protein
	FJ661_07220	sugar nucleotide-binding protein
	rfbB	dTDP-glucose 4,6-dehydratase
	FJ661_07210	glycosyltransferase
	FJ661_07205	polysaccharide biosynthesis protein
	FJ661_07200	glycosyltransferase family 2 protein
	FJ661_07195	DUF2304 domain-containing protein
	FJ661_07190	glycosyltransferase
	FJ661_07185	glycosyltransferase
	rfbA	glucose-1-phosphate thymidylyltransferase RfbA
	FJ661_07175	hypothetical protein
	FJ661_06190	phosphoglucosamine mutase
	rpsI	30S ribosomal protein S9
	rplM	50S ribosomal protein L13
	FJ661_06175	prepilin peptidase
	FJ661_06170	Flp family type IVb pilin
	FJ661_06165	pilus assembly protein
	FJ661_06160	hypothetical protein
	FJ661_06155	pilus assembly protein CpaB
	FJ661_06150	MinD/ParA family protein
	FJ661_06145	CpaF family protein
	FJ661_06140	type II secretion system protein F
	FJ661_06135	type II secretion system F family protein
	FJ661_06130	Flp family type IVb pilin
	FJ661_06125	pilus assembly protein
	FJ661_06120	pilus assembly protein TadG
	FJ661_06115	flagellar biosynthesis protein FlgA
	FJ661_06110	MinD/ParA family protein
	FJ661_04710	hypothetical protein
	FJ661_04705	hypothetical protein
	FJ661_04700	polysaccharide biosynthesis tyrosine autokinase
	FJ661_04695	ISL3 family transposase
	pseI	pseudaminic acid synthase
	FJ661_04685	hypothetical protein

	FJ661_04680	glycosyl hydrolase
	FJ661_04675	glycosyltransferase
	FJ661_04670	glycosyltransferase
	FJ661_04665	hypothetical protein
	pseB	UDP-N-acetylglucosamine 4,6-dehydratase (inverting)
	FJ661_04655	UDP-4-amino-4, 6-dideoxy-N-acetyl-beta-L-altrosamine transaminase
	FJ661_04650	spore coat protein
	FJ661_04645	UDP-2,4-diacetamido-2,4, 6-trideoxy-beta-L-altropyranose hydrolase
	FJ661_04640	sugar transferase
	FJ661_04635	DUF4012 domain-containing protein
	FJ661_04630	LPXTG cell wall anchor domain-containing protein
	FJ661_04625	VanZ family protein
	galU	UTP--glucose-1-phosphate uridylyltransferase GalU
	FJ661_04615	Hpt domain-containing protein
	csrA	carbon storage regulator CsrA
	FJ661_04605	hypothetical protein
	FJ661_04600	sigma-70 family RNA polymerase sigma factor
	FJ661_04595	FHIPEP family type III secretion protein
	FJ661_04590	EscU/YscU/HrcU family type III secretion system export apparatus switch protein
	FJ661_04740	response regulator transcription factor
	FJ661_04735	hypothetical protein
	FJ661_04730	signal peptidase I
	FJ661_04725	hypothetical protein
	FJ661_04720	MinD/ParA family protein
	FJ661_04715	winged helix-turn-helix transcriptional regulator
	FJ661_04710	hypothetical protein
	FJ661_04705	hypothetical protein
	FJ661_04700	polysaccharide biosynthesis tyrosine autokinase
	FJ661_04695	ISL3 family transposase
	pseI	pseudaminic acid synthase
	FJ661_04685	hypothetical protein
	FJ661_04680	glycosyl hydrolase
	FJ661_04675	glycosyltransferase
	FJ661_04670	glycosyltransferase
	FJ661_04665	hypothetical protein
	pseB	UDP-N-acetylglucosamine 4,6-dehydratase (inverting)
	FJ661_04655	UDP-4-amino-4, 6-dideoxy-N-acetyl-beta-L-altrosamine transaminase
	FJ661_04650	spore coat protein
	FJ661_04645	UDP-2,4-diacetamido-2,4, 6-trideoxy-beta-L-altropyranose hydrolase
	FJ661_04640	sugar transferase
	FJ661_04635	DUF4012 domain-containing protein

	FJ661_04630	LPXTG cell wall anchor domain-containing protein
	FJ661_04625	VanZ family protein
	galU	UTP--glucose-1-phosphate uridylyltransferase GalU
	FJ661_04615	Hpt domain-containing protein
	csrA	carbon storage regulator CsrA
	FJ661_04605	hypothetical protein
	FJ661_04600	sigma-70 family RNA polymerase sigma factor
	FJ661_04595	FHIPEP family type III secretion protein
	FJ661_04590	EscU/YscU/HrcU family type III secretion system export apparatus switch protein
	FJ661_04585	flagellar biosynthetic protein FliR
	fliQ	flagellar biosynthesis protein FliQ
	fliP	flagellar type III secretion system pore protein FliP
	FJ661_04570	flagellar biosynthetic protein FliO
	fliN	flagellar motor switch protein FliN
	FJ661_04560	hypothetical protein
	FJ661_04555	flagellar motor protein MotB
	FJ661_04550	motility protein A
	FJ661_04545	hypothetical protein
	FJ661_04540	flagellar hook protein FlgE
	FJ661_04535	flagellar hook capping protein
	FJ661_04530	flagellar hook-length control protein FliK
	FJ661_04525	NlpC/P60 family protein
	FJ661_04520	flagellar export protein FliJ
	FJ661_04515	FliI/YscN family ATPase
	FJ661_04510	hypothetical protein
	fliG	flagellar motor switch protein FliG
	fliF	flagellar M-ring protein FliF
	fliE	flagellar hook-basal body complex protein FliE
	FJ661_04490	flagellar basal-body rod protein FlgC
	FJ661_04485	flagellar biosynthesis protein FlgB
	FJ661_04480	hypothetical protein
	FJ661_10705	FAD-binding oxidoreductase
	FJ661_10710	L-lactate permease
	FJ661_10715	GntR family transcriptional regulator
	FJ661_10720	alpha/beta hydrolase
	FJ661_10725	enoyl-ACP reductase
	metH	methionine synthase
	FJ661_10735	hypothetical protein
	FJ661_10740	hypothetical protein
	FJ661_10745	hypothetical protein
	FJ661_10750	hypothetical protein

	FJ661_10755	ABC transporter ATP-binding protein
	FJ661_10760	ABC transporter ATP-binding protein
	FJ661_10765	hydrolase
	FJ661_10770	substrate-binding domain-containing protein
	FJ661_10775	gluconate 2-dehydrogenase subunit 3 family protein
	FJ661_10780	GMC family oxidoreductase
	FJ661_10785	aldo/keto reductase
	FJ661_10790	ABC transporter permease
	FJ661_14510	helix-turn-helix transcriptional regulator
	FJ661_14515	sugar transferase
	FJ661_14520	Gfo/Idh/MocA family oxidoreductase
	FJ661_14525	NAD-dependent epimerase/dehydratase family protein
	FJ661_14530	DegT/DnrJ/EryC1/StrS family aminotransferase
	FJ661_14535	acetyltransferase
	FJ661_14540	glycosyltransferase family 2 protein
	FJ661_14545	glycosyltransferase
	FJ661_14550	lipopolysaccharide biosynthesis protein
	FJ661_14555	DegT/DnrJ/EryC1/StrS family aminotransferase
	FJ661_14560	glycosyltransferase family 2 protein
	FJ661_14565	hypothetical protein
	FJ661_14570	O-antigen ligase domain-containing protein
	FJ661_14575	hypothetical protein
	FJ661_14580	hypothetical protein
	FJ661_14585	hypothetical protein
	FJ661_14590	WecB/TagA/CpsF family glycosyltransferase
	FJ661_14595	hypothetical protein
	FJ661_14600	DUF4082 domain-containing protein
	FJ661_14915	nitrate reductase
	FJ661_14920	hypothetical protein
	FJ661_14925	glyoxalase
	FJ661_14930	hypothetical protein
	FJ661_14935	LysR family transcriptional regulator
	FJ661_20655	phage holin family protein
	FJ661_20660	hypothetical protein
	FJ661_20310	recombinase family protein
	FJ661_20305	hypothetical protein
	FJ661_20300	hypothetical protein
	FJ661_20295	DUF91 domain-containing protein
	FJ661_20290	HNH endonuclease
	FJ661_20285	GIY-YIG nuclease family protein
	FJ661_20280	ATP-binding protein

	FJ661_20275	hypothetical protein
	FJ661_20270	DNA methyltransferase
	FJ661_20265	DUF262 domain-containing protein
	FJ661_20260	helix-turn-helix domain-containing protein
	FJ661_20255	hypothetical protein
	FJ661_20250	YfbU family protein
	FJ661_20245	hypothetical protein
	FJ661_20240	hypothetical protein
	FJ661_20235	ATP-dependent DNA ligase
	FJ661_20245	hypothetical protein
	FJ661_20240	hypothetical protein
	FJ661_20235	ATP-dependent DNA ligase
	FJ661_20230	hypothetical protein
	FJ661_20225	hypothetical protein
	FJ661_20220	hypothetical protein
	FJ661_20215	DUF4192 domain-containing protein
	FJ661_20210	IS110 family transposase
	FJ661_20205	hypothetical protein
	FJ661_20200	hypothetical protein
	FJ661_20195	hypothetical protein
	FJ661_20190	GNAT family N-acetyltransferase
	FJ661_20185	hypothetical protein
	FJ661_20180	hypothetical protein
	FJ661_20175	hypothetical protein
	FJ661_20170	hypothetical protein
	FJ661_20165	hypothetical protein
	FJ661_20160	alpha/beta hydrolase
	FJ661_20155	hypothetical protein
	FJ661_20150	DUF2199 domain-containing protein
	FJ661_20145	SRPBCC family protein
	FJ661_20140	recombinase family protein
	dprA	DNA-protecting protein DprA
	FJ661_20680	pyruvate dehydrogenase (acetyl-transferring), homodimeric type
	FJ661_20635	Lsr2 family protein
	FJ661_20570	DUF4192 domain-containing protein
	FJ661_20575	hypothetical protein
	hflB	ATP-dependent zinc metalloprotease FtsH
	FJ661_20555	SGNH/GDSL hydrolase family protein
	FJ661_20590	phage holin family protein
	FJ661_20595	DUF3618 domain-containing protein
	FJ661_20690	pyruvate dehydrogenase (acetyl-transferring), homodimeric type

	FJ661_19445	(2Fe-2S)-binding protein
	FJ661_19450	xanthine dehydrogenase family protein subunit M
	FJ661_19455	CdaR family transcriptional regulator
	FJ661_19460	CdaR family transcriptional regulator
	FJ661_19465	DUF861 domain-containing protein
	FJ661_19470	alpha/beta hydrolase
	FJ661_19475	xanthine dehydrogenase family protein molybdopterin-binding subunit
	FJ661_19480	Dabb family protein
	FJ661_19485	cyclase family protein
	FJ661_19490	2,6-dihydroxypyridine 3-hydroxylase
	FJ661_19495	carbon monoxide dehydrogenase
	FJ661_19500	carbon-nitrogen family hydrolase
	FJ661_19505	MoxR family ATPase
	FJ661_19510	VWA domain-containing protein
	FJ661_19515	XdhC family protein
	FJ661_19520	hypothetical protein
	FJ661_19525	nucleotidyltransferase family protein
	FJ661_19530	PucR family transcriptional regulator
	FJ661_19535	APC family permease
	FJ661_19540	heavy metal-responsive transcriptional regulator
	merA	mercury(II) reductase
	FJ661_19550	AAA family ATPase
	FJ661_19555	IS21 family transposase
	purU	formyltetrahydrofolate deformylase
	FJ661_19565	FAD-dependent oxidoreductase
	FJ661_19570	bifunctional methylenetetrahydrofolate dehydrogenase/methenyltetrahydrofolate cyclohydrolase
	FJ661_19575	DUF2437 domain-containing protein
	FJ661_19580	NAD-dependent succinate-semialdehyde dehydrogenase
	FJ661_19530	PucR family transcriptional regulator
	FJ661_19535	APC family permease
	FJ661_19540	heavy metal-responsive transcriptional regulator
	merA	mercury(II) reductase
	FJ661_19550	AAA family ATPase
	FJ661_19555	IS21 family transposase
	purU	formyltetrahydrofolate deformylase
	FJ661_19565	FAD-dependent oxidoreductase
	FJ661_19570	bifunctional methylenetetrahydrofolate dehydrogenase/methenyltetrahydrofolate cyclohydrolase
	FJ661_19575	DUF2437 domain-containing protein
	FJ661_19580	NAD-dependent succinate-semialdehyde dehydrogenase
	FJ661_19585	FAD-dependent oxidoreductase
	FJ661_19590	DUF861 domain-containing protein

	FJ661_19595	NAD(P)H-dependent oxidoreductase
	FJ661_19600	hypothetical protein
	FJ661_19605	hypothetical protein
	FJ661_19610	betaine/proline/choline family ABC transporter ATP-binding protein
	FJ661_19615	ABC transporter permease subunit
	FJ661_19620	hypothetical protein
	FJ661_19625	hypothetical protein
	FJ661_19630	site-specific integrase
	FJ661_19635	helix-turn-helix transcriptional regulator
	FJ661_19640	hypothetical protein
	FJ661_19645	META domain-containing protein
	FJ661_19650	hypothetical protein
	FJ661_19655	hypothetical protein
	FJ661_19660	KfrA protein
	FJ661_19665	helix-turn-helix domain-containing protein
	FJ661_19670	hypothetical protein
	FJ661_19675	relaxase
	FJ661_20435	recombinase family protein
	FJ661_20440	DDE-type integrase/transposase/recombinase
	FJ661_20445	ATP-binding protein
	FJ661_20450	hypothetical protein
	FJ661_20455	STAS/SEC14 domain-containing protein
	FJ661_20460	GAF and ANTAR domain-containing protein
	FJ661_20465	DUF2905 domain-containing protein
	FJ661_20470	DUF3040 domain-containing protein
	FJ661_19705	transposase
	FJ661_19710	hypothetical protein
	FJ661_19715	hypothetical protein
	FJ661_19720	hypothetical protein
	FJ661_19725	hypothetical protein
	FJ661_19730	DNA cytosine methyltransferase
	FJ661_19735	sigma-70 family RNA polymerase sigma factor
	FJ661_19740	hypothetical protein
	FJ661_19745	hypothetical protein
	FJ661_19750	hypothetical protein
	FJ661_19755	hypothetical protein
	FJ661_19760	ParB/RepB/Spo0J family partition protein
	FJ661_19765	hypothetical protein
	FJ661_19770	MobC family plasmid mobilization relaxosome protein
	FJ661_19775	relaxase
	FJ661_19780	hypothetical protein

	FJ661_19805	type IV secretory system conjugative DNA transfer family protein
	FJ661_19810	ATP-binding protein
	FJ661_19815	hypothetical protein
	FJ661_19820	hypothetical protein
	FJ661_19825	hypothetical protein
	FJ661_19830	hypothetical protein
	FJ661_19835	hypothetical protein
	FJ661_19840	chromosome partitioning protein ParA
	FJ661_19845	hypothetical protein
	FJ661_19850	M23 family metallopeptidase
	FJ661_19855	helix-turn-helix transcriptional regulator
	FJ661_19860	hypothetical protein
	FJ661_19865	helix-turn-helix domain-containing protein
	FJ661_19870	ParA family protein
	FJ661_19875	hypothetical protein
	FJ661_19880	AsnC family protein
	FJ661_19885	recombinase family protein
	FJ661_19890	helicase
	FJ661_19895	hypothetical protein
	FJ661_19900	hypothetical protein
	FJ661_19905	recombinase family protein
	FJ661_19910	hypothetical protein
	FJ661_19915	alpha/beta hydrolase
	FJ661_19920	GNAT family N-acetyltransferase
	FJ661_19925	IS256 family transposase
	FJ661_20675	hypothetical protein
	FJ661_20560	IS21 family transposase
	FJ661_20565	ATP-binding protein
	FJ661_20695	hypothetical protein
	FJ661_20685	pyruvate dehydrogenase (acetyl-transferring), homodimeric type
	lipA	lipoyl synthase
	FJ661_20665	pyruvate dehydrogenase (acetyl-transferring), homodimeric type
	FJ661_20700	hypothetical protein
	FJ661_20475	DUF3040 domain-containing protein
	FJ661_20480	DUF2905 domain-containing protein
	FJ661_20685	pyruvate dehydrogenase (acetyl-transferring), homodimeric type
	lipA	lipoyl synthase
	FJ661_20665	pyruvate dehydrogenase (acetyl-transferring), homodimeric type
	FJ661_20700	hypothetical protein
	FJ661_20475	DUF3040 domain-containing protein
	FJ661_20480	DUF2905 domain-containing protein

	FJ661_20485	GAF and ANTAR domain-containing protein
	FJ661_20490	heavy metal-responsive transcriptional regulator
	merA	mercury(II) reductase
	FJ661_20500	AAA family ATPase
	FJ661_20505	IS21 family transposase
	FJ661_20510	DNA replication protein
	FJ661_20580	ISL3 family transposase
	FJ661_20670	Hsp20/alpha crystallin family protein
	FJ661_20605	IS21 family transposase
	FJ661_20610	DNA replication protein
	FJ661_20615	Lsr2 family protein
	FJ661_20645	DNA replication protein
	hflB	ATP-dependent zinc metalloprotease FtsH
	FJ661_20545	SGNH/GDSL hydrolase family protein
	FJ661_19930	hypothetical protein
	FJ661_19935	DUF948 domain-containing protein
	hflB	ATP-dependent zinc metalloprotease FtsH
	FJ661_20545	SGNH/GDSL hydrolase family protein
	FJ661_19930	hypothetical protein
	FJ661_19935	DUF948 domain-containing protein
	FJ661_19940	(S)-6-hydroxynicotine oxidase
	FJ661_19945	xanthine dehydrogenase family protein molybdopterin-binding subunit
	FJ661_19950	(2Fe-2S)-binding protein
	FJ661_19955	xanthine dehydrogenase family protein subunit M
	FJ661_19960	MoaD/ThiS family protein
	moaA	GTP 3',8-cyclase MoaA
	FJ661_19970	molybdopterin molybdotransferase MoeA
	moaC	cyclic pyranopterin monophosphate synthase MoaC
	FJ661_19980	molybdenum cofactor biosynthesis protein MoaE
	modA	molybdate ABC transporter substrate-binding protein
	modB	molybdate ABC transporter permease subunit
	FJ661_19995	ABC transporter ATP-binding protein
	FJ661_20000	hypothetical protein
	FJ661_20005	glycoside hydrolase family 3 protein
	FJ661_20010	MFS transporter
	FJ661_20015	MFS transporter
	FJ661_20020	hypothetical protein
	FJ661_20025	IS630 family transposase
	FJ661_20030	helix-turn-helix domain-containing protein
	FJ661_20035	hypothetical protein
	FJ661_20040	hypothetical protein

	FJ661_20045	DUF4913 domain-containing protein
	FJ661_20050	conjugal transfer protein

Table 3. Comparative studies of genes responsible for endophytic behaviour. sp. 638 is *Enterobacter* sp. 638 , MHSD1 is *Pseudarthrobacter phenanthrenivorans* MHSD1, Sphe3 is *Pseudarthrobacter phenanthrenivorans* Sphe3. A (-) represents the absence of genes responsible for endophytic behaviour and (+) indicated the presence of gene in the genome. The table was adapted from studies done by Ali *et al*. (2014)

Function		sp. 638	MHSD1	Sphe3
Transport	Lysine exporter protein lyse/ygga	+	+	+
	Major facilitator superfamily protein	-	+	+
	Branched-chain amino acid ABC transporter ATPase	+	-	-
	Branched-chain amino acid ABC transporter inner membrane protein	+	-	-
	NAD(P)(+) transhydrogenase (AB-specific)	-	+	+
	ABC transporter related	+	+	+
	Metabolite/Hþ symporter, major facilitator superfamily (MFS)	-	-	-
	Extracellular solute-binding protein family 1	-	+	+
	Gluconate 2-dehydrogenase (acceptor)	-	+	+
	Gluconate 2-dehydrogenase (acceptor)	-	+	+
	Gluconate 2-dehydrogenase (acceptor)	-	+	+
Secretion and delivery system	Type VI secretion protein, VC_A0107 family	-	-	-
	Type VI secretion protein, EvpB/VC_A0108 family	-	-	-
	Type VI secretion ATPase,ClpV1family	-	-	-
	RND family efflux transporter MFP subunit	+	-	-
Phyto-functional assays	Phosphorus solubilisation	-	-	-

	IAA (indole-3-acetic acid) production	-	-	-
	ACC (1-aminocyclopropane-1-carboxylate) deaminase	-	-	-
	Siderophore production	+	+	+
	Nitrogen fixation	+	+	-
Plant polymer degradation/ modification	Alpha/beta hydrolase fold	-	-	+
	Alpha, alpha-trehalase	+	-	-
	Cupin	+	+	+
	Peptidase M48 Ste24p	+	-	-
Transcriptional regulator	Asnc family transcriptional regulator	-	+	+
	Regulator protein FrmR	+	-	-
	AraC family transcriptional regulator	-	-	-
	Two component transcriptional regulator , winged helix family	-	+	+
	Transcriptional regulator, DeoR family	-	+	+
	Transcriptional regulator, LysR family	-	+	+
	Transcriptional regulator ,LytR family	+	+	-
	LrgB family protein	-	-	-
	Flavoprotein WrbA	+	-	-
Detoxification	Glutathione S-transferase	+	+	+
	Short-chain dehydrogenase/reductase SDR	+	-	-
	S-(hydroxymethyl) glutathione dehydrogenase/class III alcohol dehydrogenase	-	-	-
	2-dehydropantoate2-reductase	+	-	-
Redox potential maintenance	Acetoacetyl-coa reductase	-	-	-
	Acetaldehyde dehydrogenase	-	-	-
	Carbonate dehydratase	+	-	-
	Aldehyde dehydrogenase	-	+	+
	Malate/L-lactate dehydrogenase	+	+	+
	3-hydroxyisobutyrate dehydrogenase	-	+	+

Virulence and antibiotic factors	YihY/virulence factor BrkB family protein	-	+	+
	Polyketide antibiotic transporter	-	-	+
	Antibiotic biosynthesis monooxygenase	+	+	+
Other	2-isopropylmalate synthase	+	+	+
	Diaminopimelate decarboxylase	+	+	+
